# Odor Aversion and Pathogen-Removal Efficiency in Grooming Behavior of the Termite *Coptotermes formosanus*

**DOI:** 10.1371/journal.pone.0047412

**Published:** 2012-10-15

**Authors:** Aya Yanagawa, Nao Fujiwara-Tsujii, Toshiharu Akino, Tsuyoshi Yoshimura, Takashi Yanagawa, Susumu Shimizu

**Affiliations:** 1 Research Institute for Sustainable Humanosphere, Kyoto University, Uji City, Kyoto, Japan; 2 National Institute of Agrobiological Science, Ohwashi City, Tsukuba, Japan; 3 Department of Applied Biology, Kyoto Institute of Technology, Kyoto City, Kyoto, Japan; 4 Biostatistics Centre, Kurume University, Kurume city, Fukuoka, Japan; 5 Institute of Biological Control, Kyushu University, Fukuoka City, Fukuoka, Japan; Barnard College, Columbia University, United States of America

## Abstract

The results of biocontrol with entomopathogens in termites have been discouraging because of the strong social hygiene behavior for removing pathogens from termite colonies. However, the mechanism of pathogen detection is still unclear. For the successful application of biopesticides to termites in nature, it would be beneficial to identify substances that could disrupt the termite’s ability to perceive pathogens. We hypothesized that termites can perceive pathogens and this ability plays an important role in effective hygiene behavior. In this study, pathogen-detection in the subterranean termite *Coptotermes formosanus* was investigated. We performed quantitative assays on conidia removal by grooming behavior using epifluoresence microscopy and Y-maze tests to examine the perception of fungal odor by termites. Three species each of high- and low-virulence entomopathogenic fungi were used in each test. The results demonstrated that termites removed conidia more effectively from a nestmate’s cuticle if its odor elicited stronger aversion. Highly virulent pathogens showed higher attachment rates to termite surfaces and their odors were more strongly avoided than those of low-virulence isolates in the same species. Moreover, termites appeared to groom each other more persistently when they had more conidia on their bodies. In brief, insect perception of pathogen-related odor seems to play a role in the mechanism of their hygiene behavior.

## Introduction

*Coptotermes formosanus* is one of the most destructive insects for houses and other wood structures [1], [2]. While various termiticidal chemicals have been used for termite control, their extensive use can cause significant environmental hazards [3], [4]. Entomopathogenic fungi have been proposed as an alternative agent for termite control [5]–[8], but attempts to apply them have revealed difficulties in the use of fungi to control termites [9]. One reason for these failures is the uncertainty regarding the influence of termite hygiene behaviors during infection by pathogenic fungi [10]. Although grooming is a well-documented social hygiene behavior in termites that enhances colony health [11]–[15], as pointed out by Roy et al. [16], its role and mechanism in the elimination of microbes has not yet been fully described. It is important to understand how insects perceive pathogens that elicit grooming behavior since this information could promote the successful application of such pathogens. In general, while termites are susceptible to many different fungi at an individual level, different isolates from the same species can exhibit a range of virulence [17], [18]. In this study, we compared termite grooming behavior against high- and low-virulence fungi. Isolates were selected from three species, *Metarhizium, Isaria* and *Beauveria,* based on previous data [19]. All of these species are well known as biological control agents of pest insects [20], [21]. Conidia attachment and persistence on the insect surface were compared with regard to fungal virulence. Furthermore, termite reactions related to olfactory perception were examined in a Y-maze test to see whether a musty odor had any effect on grooming behavior. We postulated that the perception of pathogen-related odor regulates pathogen-resistant hygiene behaviors. In this study, the effect of odor on pathogen-removal from the body surface was examined together with information on the chemical constituents of the odor substance as identified by gas chromatography and mass spectrometry (GC/MS).

## Results

### Effect of Population Density on Termite Susceptibility

Table 1 shows the virulence data (LD_50_ values) of colony C for the six entomopathogenic fungi toward termites reared as individuals compared to those reared in groups. For highly virulent fungi, termite resistance increased drastically with an increase in the population density. The LD_50_ values for low-virulence isolates depended less on the group size. The results in colonies A and B showed similar trends (Table S1).

### Detection of Conidia on Termite Cuticles

**Figure 1:**
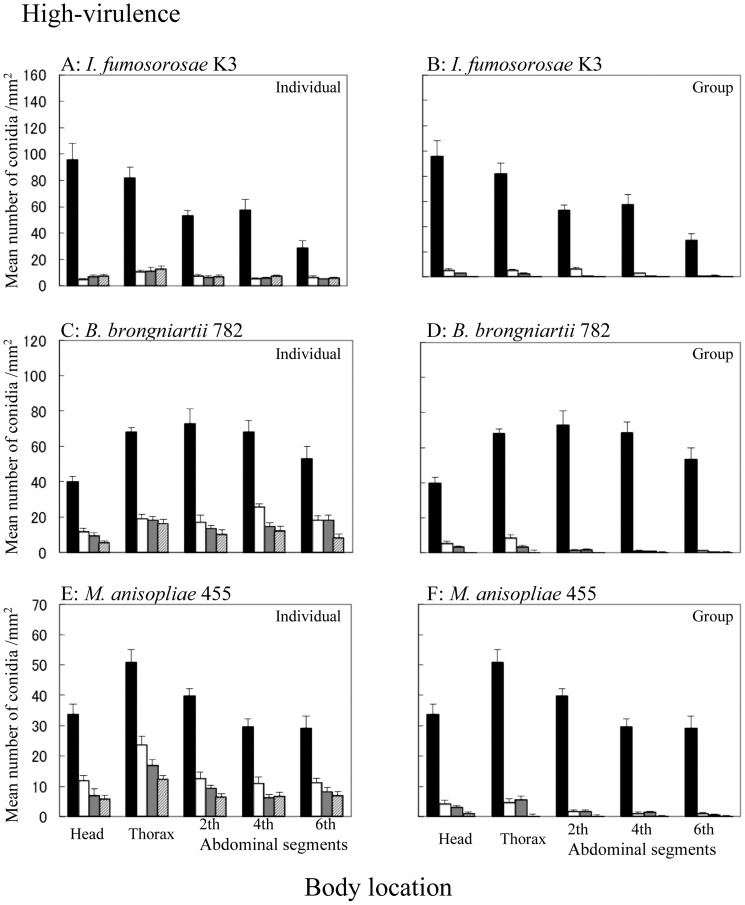
Attachment and persistence of FITC-labeled conidia of high-virulence entomopathogenic fungi on *C. formosanus* cuticle. A: Termites treated with *I. fumosorosea* K3 were reared individually (y = −1.281x + 31.90, r^2^ = 0.160, p<0.001, linear regression). B: Termites treated with *I. fumosorosea* K3 were reared as a group (y = −1.553x + 30.01, r^2^ = 0.209, p<0.001, linear regression). C: Termites treated with *B. brongniartii* 782 were reared individually (y = −1.319x + 36.85, r^2^ = 0.266, p<0.001, linear regression). D: Termites treated with *B. brongniartii* 782 were reared as a group (y = −1.474x + 28.67, r^2^ = 0.247, p<0.001, linear regression). E: Termites treated with *M. anisopliae* 455 were reared individually (y = −0.789x + 23.51, r^2^ = 0.262, p<0.001, linear regression). F: Termites treated with *M. anisopliae* 455 were reared as a group (y = −0.900x + 17.98, r^2^ = 0.257, p<0.001, linear regression). n = 10. : Termites just after inoculation. : Termites at 3 h post-inoculation. : Termites at 6 h post-inoculation. : Termites at 24 h post-inoculation. Bars at the top of the columns represent standard errors. n = 10.

The attachment and persistence of conidia on the termite surface were estimated by the use of FITC-labeled conidia. The results are illustrated in Figs. 1 and S1 with respect to population densities, times and sites. The removal pattern differed according to the fungal virulence and isolate (p<0.001 for virulence and isolate under both densities, ANOVA). When termites were inoculated with highly-virulent fungal isolates, the initial attachments of conidia were 10- to 100-fold greater than when they were inoculated with low-virulence fungal isolates (Figs. 1 and S1). A significant difference was observed between single termites and 10-termite groups for highly virulent fungal isolates (*I. fumosorosea* K3: p = 0.003, *B. brongniartii* 782: p<0.001 and *M. anisopliae* 455: p<0.001, Table S2 in density parameter), while no significant difference was observed between the two population densities for low-virulence fungal isolates (*I. fumosorosea* 8555: p = 0.868, *B. bassiana* F1214: p = 0.641 and *M. anisopliae* UZ: p = 0.460, Table S3 in density parameter). Furthermore, the conidia of highly virulent isolates were removed 2 to 10 times quicker than those of low-virulence isolates in each genus (Figs. 1 and S1, see coefficient of linear regression). The removal of conidia from the individual/nestmate surface was significant for all of the high-virulence isolates, but was much more moderate for low-virulence isolates.

### Behavior Test Toward Fungal Odor

**Figure 2:**
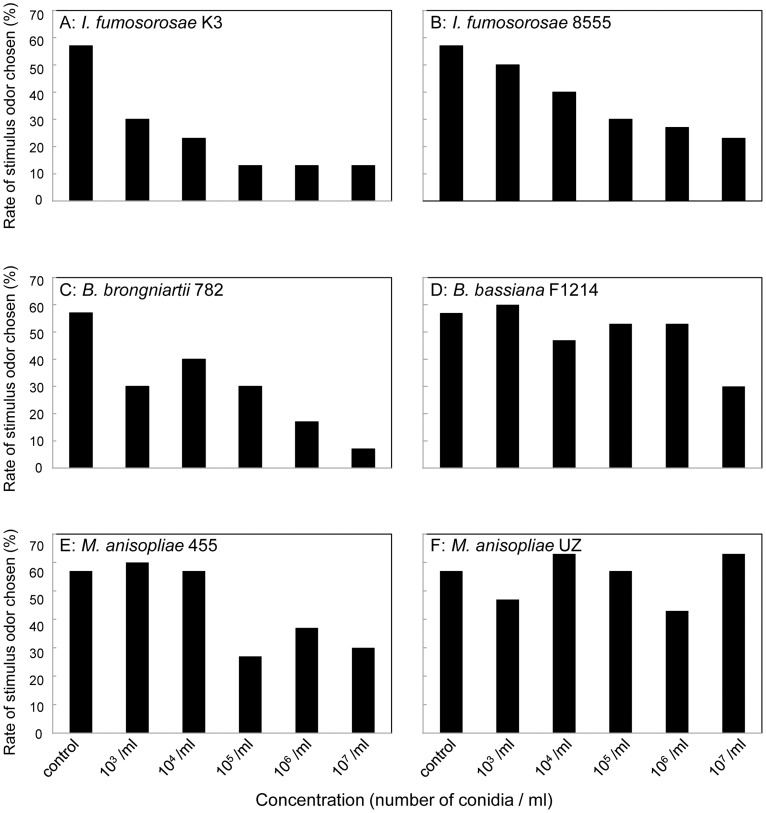
Concentration-dependent avoidance of 6 isolates of high- and low-virulence entomopathogenic fungi by the termite *C. formosanus*. The vertical axis shows the proportion of termites that chose the stimulus odor branch. n = 30. A: Choice of the odor from *I. fumosorosea* K3 conidia suspension (y = −0.067x + 0.53, r^2^ = 0.120, p<0.001, linear regression). B: Choice of the odor from *I. fumosorosea* 8555 conidia suspension (y = −0.051x + 0.60, r^2^ = 0.057, p = 0.001, linear regression). C: Choice of the odor from *B. brongniartii* 782 conidia suspension (y = −0.066x + 0.58, r^2^ = 0.106, p<0.001, linear regression). D: Choice of the odor from *B. bassiana* F1214 conidia suspension (y = −0.028x + 0.62, r^2^ = 0.016, p = 0.088, linear regression). E: Choice of the odor from *M. anisopliae* 455 conidia suspension (y = −0.046x + 0.64, r^2^ = 0.044, p = 0.005, linear regression). F: Choice of the odor from *M. anisopliae* UZ conidia suspension (y = 0.002x + 0.54, r^2^<0.001, p = 0.922, linear regression).

Odor perception and preference were examined by a Y-maze two-choice test. The data from all 3 colonies were pooled since there was no significant difference in the choice of branches among the colonies (p = 0.205, ANOVA). The termite choice of the control/fungal-odor branches is illustrated in Fig. S2. Termites significantly avoided the odors of all isolates except *M. anisopliae* UZ, which has relatively low virulence. The concentration-dependent avoidance of fungal odor significantly varied depending on the fungal isolate (p<0.001, ANOVA). Stratification of the isolates according to virulence demonstrated that termites showed stronger concentration-dependent avoidance of the odor of high-virulence isolates than the odor of low-virulence isolates (Fig. 2, p = 0.001, stratification analysis of a logistic model). In *Isaria* and *Beauveria,* termite aversion to fungal odor increased gradually with an increase in concentration. In *Metarhizium*, termites showed aversion to the odor of *M. anisopliae* 455 above a conidia concentration of 10^5^/ml (p<0.001, ANOVA), and there was no further increase in aversion above that concentration (p = 0.703, ANOVA). Termites were not influenced by the odor of *M. anisopliae* UZ.

### Estimation of Fungal Volatiles

**Figure 3:**
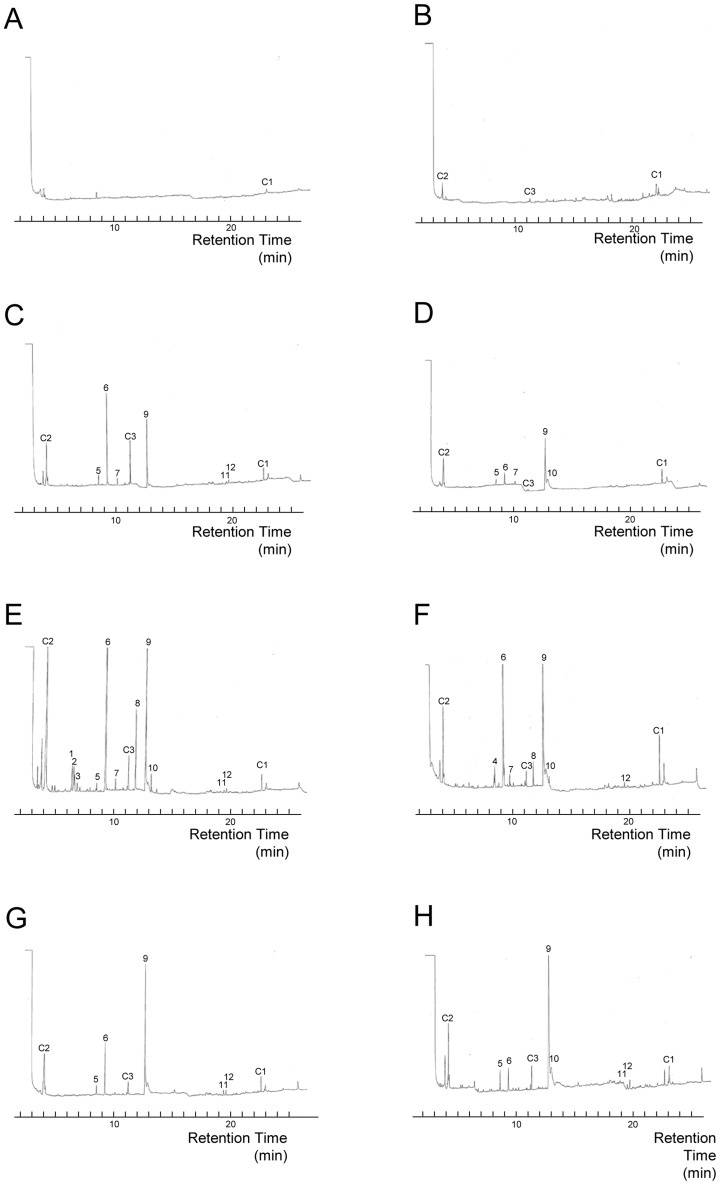
Gas chromatogram of volatiles from entomopathogenic fungi. Gas chromatogram of volatiles from A: an empty glass container. B: a glass container containing control solution L. C: a glass container containing *I. fumosorosea* K3 odor-solution. D: a glass container containing *I. fumosorosea* 8555 odor-solution. E: a glass container containing *B. brongniartii* 782 odor-solution. F: a glass container containing *B. bassiana* F1214 odor-solution. G: a glass container containing *M. anisopliae* 455 odor-solution. H: a glass container containing *M. anisopliae* UZ odor-solution. Peak number 1: RT = 6.4 min, 2: RT = 6.5 min, 3: RT = 6.6 min, 4∶8.5 min, 5: RT = 8.7 min, 6: RT = 9.3 min, 7: RT = 10.1 min, 8: RT = 11.8 min, 9: RT = 12.7 min, 10: RT = 13.0 min, 11: RT = 19.4 min, 12: RT = 19.7 min. Peaks C1, C2 and C3 indicate unknown chemicals detected in control preparations.

When we excluded 3 peaks that were seen in the controls, 12 substances were estimated to have arisen from entomopathogenic fungi (Fig. 3). 3-Methyl-1-butanol (Retention Time [RT] = 8.5 min), 3-octanone (RT = 9.3 min), 1-octen-3-ol (RT = 12.7 min) and 3-octanol (RT = 11.8 min) were identified by comparison to the mass spectra and retention times of the authentic compounds. The most common chemical, which all fungi possessed, was 1-octen-3-ol. The mass spectra indicated that 3 methyl-1-butanol was found only in *B. bassiana* F1214, while a peak with a similar RT (RT = 8.7 min, peak number 1 in Fig. 3) in other isolates was an unknown substance. The substances at RT = 6.5 min and 6.6 min (peak numbers 7 and 8 in Fig. 3) were seen only in *B. brongniartii* 782. The odor from *Beauveria* was the only odor that contained 3-octanol (peak number 9 in Fig. 3), and *Beauveria* isolates and *I. fumosorosea* 8555 shared an unknown chemical at RT = 13.0 min (peak number 5 in Fig. 3). While GC/MS analyses detected at least 40 compounds from the 6 fungal isolates, most were unknown chemicals (Fig. 3). From one culture, approximately 1–10 ng/ml substance was collected for each peak (Table S4). With regard to the identified chemicals, termites showed significant aversion to the odor of 3-octanone and a preference for 3-octanole at lower concentrations. They were indifferent to the odors of 1-octen-3-ol and 3-methyl-1-butanol (Fig. S3).

## Discussion

The effects of virulence on the interaction between the removal of conidia of entomopathogenic fungi from nestmate cuticles and the termite preference for their odors were examined in *C. formosanus*. Termites were more efficient at removing conidia of virulent isolates. At the same time, they removed conidia that had a more repellent odor. Gao et al. [22] indicated that the level of individual immunity against pathogenic infection was not related to the group size. Thus, hygiene behavior may be the most crucial factor in determining termite resistance at the group level.

We found that low-virulence conidia had a weaker attachment ability than highly virulent isolates, and termites were not as persistent in their attempts to remove those with low virulence. According to Clarkson and Charnley [23], fungal conidia should not drop off or be easily removed after they have attached to the host cuticle, since attachment is the initial event in fungal invasion of the host. Highly virulent fungal conidia show a strong ability to attach to the host insect surface [24], [25], and in termites, conidia are removed by mutual grooming behavior after this initial infection event (Fig. 1). The decrease in low -virulence conidia from the termite surface appeared to be due to natural dropping-off, rather than mutual grooming behavior, since no significant difference was observed in the decrease in conidia according to the number of nestmates in a group (Table S3, density and time parameter). Since the conidia of entomopathogenic fungi have the same shape and size at the species level, the differences in the attachment of fungi with different levels of virulence should be due to the surface structure of conidia and its interactions with the insect surface [24]–[31].

Termites showed strong aversion toward the odor of highly virulent conidia, which they removed more efficiently from the cuticles of their nestmates (Figs. 1, 2 and S1). This result suggests that odor helps termites to recognize foreign organisms that should be removed from their nestmates. Other reports, which suggested that more virulent isolates could repel termites more strongly [32], [33], also support this finding. On the other hand, an increase in grooming behavior induced by a musty odor occurred independent of virulence [34]. In addition, the termite aversion to fungal odor gradually increased with an increase in the conidia concentration in the high-virulence isolates of *Beauveria* and *Isaria.* In contrast, termites started to show aversion to *M. anisopliae* 455 beginning at a concentration of 10^5^/ml and this aversion did not increase with a further increase in the concentration (Fig. 2). On the other hand, termites did not react to the odor of *M. anisopliae* UZ (Figs. 2 and S2). Nishi et al. reported that *M. anisopliae* may be a potential fungal pathogen of termites [35]. In this study, termites were more susceptible to 2 isolates of *Metarhizium* than to other isolates (Table 1), and may have developed different behaviors in response to familiar and unfamiliar pathogens. The interactions between a fungal virulence and the aversion of termites to its odor require further careful investigation.

**Table 1:** Virulence of entomopathogenic fungi to the termite from colony C.

Fungus	LD_50_(CFUs/ml)		
	Number of termites/dish		
	1	5	10
**<Highly virulent isolates>**			
***I. fumosoroseus*** ** K3**	<9.6×10^3^	1.0×10^6^	5.5×10^6^
***B. brongniartii*** ** 782**	2.3×10^4^	6.2×10^5^	7.4×10^5^
***M. anisopliae*** ** 455**	<9.2×10^2^	1.8×10^4^	6.9×10^4^
**<Weak virulent isolates>**			
***I. fumosoroseus*** ** 8555**	8.3×10^5^	1.2×10^7^	2.3×10^7^
***B. bassiana*** ** F1214**	3.5×10^5^	2.8×10^5^	5.5×10^5^
***M. anisopliae*** ** UZ**	<2.5×10^3^	5.6×10^5^	5.8×10^5^

The MonoTrap DCC18 allows us to easily collect volatiles and quantify chemical compounds under conditions similar to those in nature. In addition, due to the new absorption method with a MonoTrap disc, we could collect more chemicals than with SPME [34]. In this analysis, no common volatile was associated with fungal virulence or species. However the development condition of a fungus also influences its volatile composition [36], [37]. Despite uniform incubation periods, the amount of volatiles collected changed for the same isolate, and it was difficult to achieve uniform growth conditions for the fungi. In addition, in this analysis, most of the chemicals collected were unknown and difficult to identify. In a previous study, termites showed the ability to recognize fungal odor at the isolate level [38]. It should be useful to further clarify the relation between virulence and odor signals in grooming behavior.

Insect olfaction seems to help protect insects from disease [39]–[42]. This study found that while termites avoid fungi when they are at a distance, they move toward pathogens to eliminate them from their colony once the pathogens become attached. Further studies on the perception of pathogens by insects, particularly with regard to how they relate to the social behavior of the host, should help promote the use of microbial agents for termite control.

## Materials and Methods

### Insects

Mature workers of *C. formosanus* were obtained from three laboratory colonies, A, B and C, that were maintained in the dark at 28°C and at more than 85% R.H. at the Deterioration Organism Laboratory (DOL) of the Research Institute for Sustainable Humanosphere, Kyoto University, Japan. Worker termites were transferred from the respective colony into 90×15 mm Petri dishes containing a wet paper disc (Whatman No. 1) in a dark chamber at 25°C for 1 to 3 weeks before use.

### Preparation of Conidial Suspensions

Three highly virulent entomopathogenic fungi, *M. anisopliae* 455, *I. fumosorosea* K3 and *B. brongniartii* 782, and three low-virulence isolates, *M. anisopliae* UZ, *I. fumosorosea* 8555 and *B. bassiana* F1214, were selected based on previous data. Termites showed 90–100% mortality at 7 days after inoculation with highly virulent fungi and 10–50% mortality with low-virulence fungi [18].

*Metarhizium* were maintained on potato dextrose agar (potato extract, 0.4%; glucose, 2.0%; agar, 1.5%) and isolates of *Isaria* and *Beauveria* were maintained on L-broth agar (polypeptone, 1%; yeast extract, 0.3%; sucrose, 2.0%; NaCl, 0.5%; agar, 2.0%) at 25°C. Conidia were harvested with a brush from 14- to 18-day-old cultures and suspended in various solutions as described below.

To investigate the effect of population density on termite susceptibility to fungal infection, conidial suspensions were prepared in a 0.025% aqueous solution of Tween 20 (0.025% Tween 20 solution). They were diluted 10^0^ -, 10^1^ -, 10^2^ -, 10^3^ - and 10^4^ -fold (A series). The diluted suspension (0.1 ml) was pipetted onto each L-broth agar Petri dish and then spread using a sterilized glass spreader. The Petri dishes were incubated at 25°C for 3 days, and colony-forming units (CFUs/ml) were then determined from the numbers of colonies on these L-broth agar plates to calculate LD_50_. For the behavior test, conidia in 0.025% Tween 20 solution were counted with a Thoma hemocytometer (Erma INC. Tokyo) and adjusted to a concentration of 1.0×10^7^ conidia/ml (B series). For the quantitative assay, all of the conidia were preliminarily surface-labeled with 0.01% fluorescein isothiocyanate (FITC, Sigma Chemical) solution so that they could be detected on the cuticles and in the alimentary tracts of termites according to the protocols outlined by Hung and Boucias [43] (C series). FITC-labeled conidia in 0.025% Tween 20 solution were counted with a Thoma hemocytometer (Erma INC. Tokyo) and adjusted to a concentration of 1.0×10^7^ conidia/ml. To analyze the chemicals in fungal odor, conidial suspensions (D series) were prepared in 3 ml of distilled water. As many conidia as possible were harvested from one culture; 1.59×10^7^–3.94×10^8^ conidia/ml.

### Effect of Population Density on Termite Susceptibility to Fungal Infection

For inoculation, the termites were collected from the Petri dishes and placed in microcentrifuge tubes containing conidial suspensions (A series). The termites from colonies A, B and C were submerged in conidial suspensions with gentle swirling for 5 seconds and allowed to dry on a Whatman No. 1 filter paper. The termites were washed once in 0.025% Tween 20 solution to remove non-attached conidia. These termite groups were partitioned into three densities. Ten termites were placed individually in the wells of a 24-well microtiter plate containing a wet paper disc. Next, 5 termites were placed together in 35×15 mm Petri dishes containing a wet paper disc. The other termites were placed in groups of 10 in 90×15 mm Petri dishes containing a wet paper disc. Termites that were treated only with 0.025% Tween 20 solution were reared at each density as controls. They were incubated at 25°C. Seven days after inoculation, the LD_50_ was calculated according to Reed and Muench [44].

### Detection of Conidia on Termite Cuticles

Worker termites from colony C, which is the largest colony, were used in the observation of the insect surface, since the three colonies showed similar susceptibilities to fungal infection (Table S1).

Mature workers from colony C were treated with FITC-labeled conidial suspensions (C series) as described above and then washed once in 0.025% Tween 20 solution to remove non-attached conidia. They were partitioned into two densities, 1 termite and 10 termites per dish, and incubated at 25°C. At 0, 3, 6 and 24 h post-treatment, termites were sampled and stored at -20°C. The binding of FITC-labeled conidia to defined sites on the surface of termites was quantified using an epifluorescent microscope. Termites were carefully mounted in a drop of Vectashield (Vector Laboratories) to stabilize the fluorescence and examined with an epifluorescent microscope (Diaphot, Nikon, Japan) at 200×. A total of five defined sites (Head, Thorax, 2^nd^, 4^th^ and 6^th^ abdominal segments) on each termite surface were examined for the attachment of conidia. Ten termites were observed from each density.

### Behavior Test Toward Fungal Odor

The Y-maze consisted of a Y-shaped glass tube with an inner diameter of 6 mm. The single stem branch and two side branches were each 5 cm long [45]. The termites were introduced into the stem branch as described in detail below.

Stimulus air flowed from one side branch and control air flowed from the other side branch into the stem branch. The stimulus air was prepared as follows. Fresh air was taken from the outside by a diaphragm pump (AP-115 Iwaki air pump, IWAKI CO. LTD, Tokyo). Air was drawn into serially connected bottles that contained silica gel and molecular sieves 3A (1-4896-01, Shinwa Chemical Industries Ltd, Kyoto) or 5A (1-4896-02) to desiccate it. The air was then cleaned by passing it through active carbon. The cleaned air flow was divided into two channels by a Y-shaped connector. One channel was connected to a bottle (30 ml) that contained 1ml 0.025% of Tween 20 as a control and the other was connected to a bottle that contained 1 ml of a 10^7^/ml conidial suspension (B series) as a source of musty odor stimulus [38], [46]. The air channels were connected separately to the two branches of the Y-maze. The flux in each channel was regulated to be 400 ml/minute by an inline flowmeter.

One termite was placed at the inlet of the stem branch of the Y-maze and exposed to light illumination from its abdominal side to drive it to move to the branch point of the Y-maze [32]. The choice was determined when the sample termite passed the mid-point of the respective side branch (2.5 cm from the junction) from the entrance of the stem branch. First, the choice of musty odor stimulus was compared among 6 fungal isolates with a 10^7^/ml conidial suspension (B series), and the concentration-dependent reaction to each isolate was then examined. Since there was no colony-dependent difference in behavior in response to 10^7^/ml conidial suspensions (Kruskal Wallis test, p = 0.205, n = 35), worker termites from colony C were used to examine concentration-dependent reactions. Thirty repetitions were performed for each fungal isolate.

This experiment was carried out in the laboratory at 25°C under normal light conditions.

### Estimation of Fungal Volatiles

Volatile compounds derived from entomopathogenic fungal conidia were collected by a MonoTrap™ disc DCC18 (Version 1-0, GL Science Inc., Tokyo, Japan). The disc was hung 2 cm above the test solutions (D series conidial suspension) for 20 h at 35°C. Each sample consisted of 3 ml of solution, and was prepared in a glass vial. As a control solution, 3 ml of distilled water was prepared by gently washing the surface of solid L-broth agar that had not been inoculated with entomopathogenic fungi. Monotrap discs were also hung in empty glass vials to collect volatile environmental compounds as a blank control. Individual MonoTrap discs were then immersed in 1 ml dichloromethane (Nacalai Tesque, Kyoto, Japan) for elution with ultrasonication for 15 min. The desorbed solvent was concentrated to 200 µl before analysis. The quantity of adsorbed chemicals was estimated 3 times by GC/MS as described below.

Gas chromatograph (GC) analyses were conducted with a Shimadzu GC-14A equipped with a polar capillary column, DB-WAX (30m length, 0.25 mm diameter, 0.25 µm film thickness; J & W Scientific. Inc.) and a flame ionization detector. Helium was used as the carrier gas and the column head pressure was set at 2 kPa. Injection was made in split mode at 200°C. The column oven temperature was increased from 40°C to 180°C at 10°C/min and from 180°C to 220°C at 20°C/min, and kept at the final temperature for 10 min. Gas chromatograph-mass spectrometry (GC-MS) was performed using a Shimadzu QP5000 GC-MS system. The GC conditions were almost identical to those in the GC analyses, except that the column head pressure was 100 kPa. Seventy eV EI-mass spectra were recorded at a rate of 0.5s per scan. Volatile compounds were estimated by comparison of relative mass spectra and retention times with those of authentic candidate compounds, which were purchased from Nacalai Tesque (Kyoto, Japan). To confirm the volatile compounds, analyses of the chemical substances from the sample fungi were conducted several times with GC-MS using different GC columns (DB-1HT and DB-Wax), and the relative mass spectra and retention times were compared again with those of authentic compounds (Nacalai Tesque, Kyoto, Japan).

### Statistical Analysis

To compare the differences in conidia attachment and persistence on the termite surface, a Poisson regression [47] was applied to the data. For the Y-maze test, to examine the termite reaction to each fungal isolate, the Tukey-Kramer HSD test was applied. Logistic regression was used to examine the interactions between fungal virulence and the decrease in conidia from the termite body surface and between fungal virulence and the choice of branch in the Y-maze test. Analysis of variance (ANOVA) was applied to the pooled data of five defined sites on the body surface at each time interval to analyze the decrease in conidia from the termite cuticle, and the relationship could be expressed by a linear regression (y: mean number of conidia attached to five defined sites on the termite cuticle, x: time (h) post-inoculation, r^2^: Pearson’s correlation coefficient). ANOVA was also used to analyze the impact of the odor concentration on the termite choice in the Y-maze test (linear regression, y: y = 1 when a termite chose the musty odor branch, and y = 0 when a termite chose the control branch, x: concentration of conidia solution, r^2^: Pearson’s correlation coefficient). JMP 6.0 software was used for all analyses except for the Poisson regression.

## Supporting Information

Figure S1 **Attachment and persistence of FITC-labeled conidia of low-virulence entomopathogenic fungi on the cuticle of** ***C. formosanus*** **.** A: Termites treated with *I. fumosorosea* 8555 were reared individually (y = −0.014x + 1.67, r^2^ = 0.003, p = 0.451, linear regression). B: Termites treated with *I. fumosorosea* 8555 were reared as a group (y = −0.040x + 1.83, r^2^ = 0.038, p = 0.006, linear regression). C: Termites treated with *B. bassiana* F1214 were reared individually (y = −0.129x + 3.60, r^2^ = 0.136, p<0.001, linear regression). D: Termites treated with *B. bassiana* F1214 were reared as a group (y = −0.134x + 3.42, r^2^ = 0.163, p<0.001, linear regression). E: Termites treated with *M. anisopliae* UZ were reared individually (y = 0.042x + 1.68, r^2^ = 0.022, p = 0.037, linear regression). F: Termites treated with *M. anisopliae* UZ were reared as a group (y = −0.019x + 1.87, r^2^ = 0.006, p = 0.257, linear regression). : Termites just after inoculation. : Termites at 3 h post-inoculation. : Termites at 6 h post-inoculation. : Termites at 24 h post-inoculation. Bars at the top of the columns represent standard errors. n = 10.
(TIF)

Figure S2 **Choice of fungal odor branch for 6 isolates of high- and low- virulence entomopathogenic fungi.** The data obtained from colonies A, B and C were pooled. n = 100. The vertical axis shows the proportion of termites that chose the stimulus odor branch. Letters at the top of the columns indicate the results of the Tukey-Kramer HSD test.
(TIF)

Figure S3 **Concentration-dependent avoidance of fungus-related volatiles estimated by GC-MS. n = 30.** A significant change from the control response by the Wilcoxon test is indicated by asterisks* at the top of the column (***: p<0.01, **: p<0.05, *: p<0.1). A: Choice of the 3-octanone odor branch. B: 3-octanole odor branch. C: 1-octen-3-ol odor branch. D: 3-methyl-1-butanol odor branch.
(TIF)

Table S1 **Virulence of entomopathogenic fungi to the termite from colony A and colony B.**
(TIF)

Table S2 **Attachment and persistence of highly virulent conidia on the termite cuticle between the groups reared individually and reared as a group.**
(TIF)

Table S3 **Attachment and persistence of conidia on the termite cuticle between the groups reared individually and reared as a group.**
(TIF)

Table S4 **Average quantity of identified chemicals collected by MonoTrap discs in conidia suspension from one culture.**
(TIF)
