# Supplementary figures and images for: Odor Aversion and Pathogen-Removal Efficiency in Grooming Behavior of the Termite Coptotermes formosanus
Source: PLoS One. 2012 Oct 15;7(10):e47412. doi: 10.1371/journal.pone.0047412 (PMC3471821; doi:10.1371/journal.pone.0047412)

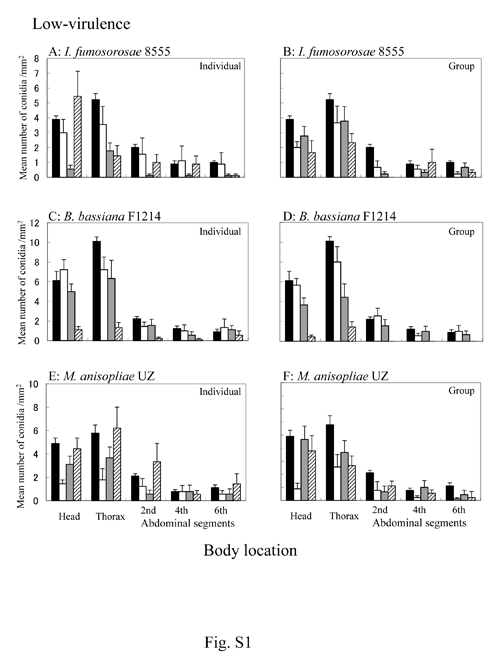

Supplement: Figure S1 — Attachment and persistence of FITC-labeled conidia of low-virulence entomopathogenic fungi on the cuticle of C. formosanus . A: Termites treated with I. fumosorosea 8555 were reared individually (y = −0.014x + 1.67, r2 = 0.003, p = 0.451, linear regression). B: Termites treated with I. fumosorosea 8555 were reared as a group (y = −0.040x + 1.83, r2 = 0.038, p = 0.006, linear regression). C: Termites treated with B. bassiana F1214 were reared individually (y = −0.129x + 3.60, r2 = 0.136, p<0.001, linear regression). D: Termites treated with B. bassiana F1214 were reared as a group (y = −0.134x + 3.42, r2 = 0.163, p<0.001, linear regression). E: Termites treated with M. anisopliae UZ were reared individually (y = 0.042x + 1.68, r2 = 0.022, p = 0.037, linear regression). F: Termites treated with M. anisopliae UZ were reared as a group (y = −0.019x + 1.87, r2 = 0.006, p = 0.257, linear regression). : Termites just after inoculation. : Termites at 3 h post-inoculation. : Termites at 6 h post-inoculation. : Termites at 24 h post-inoculation. Bars at the top of the columns represent standard errors. n = 10. (TIF) [file pone.0047412.s001.tif]

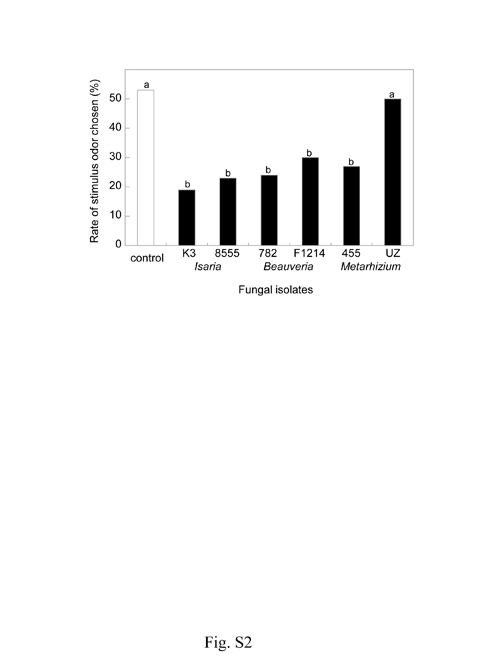

Supplement: Figure S2 — Choice of fungal odor branch for 6 isolates of high- and low- virulence entomopathogenic fungi. The data obtained from colonies A, B and C were pooled. n = 100. The vertical axis shows the proportion of termites that chose the stimulus odor branch. Letters at the top of the columns indicate the results of the Tukey-Kramer HSD test. (TIF) [file pone.0047412.s002.tif]

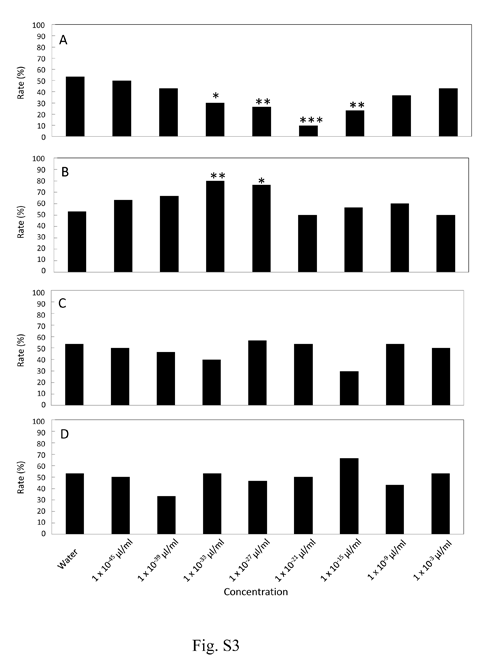

Supplement: Figure S3 — Concentration-dependent avoidance of fungus-related volatiles estimated by GC-MS. n = 30. A significant change from the control response by the Wilcoxon test is indicated by asterisks* at the top of the column (***: p<0.01, **: p<0.05, *: p<0.1). A: Choice of the 3-octanone odor branch. B: 3-octanole odor branch. C: 1-octen-3-ol odor branch. D: 3-methyl-1-butanol odor branch. (TIF) [file pone.0047412.s003.tif]

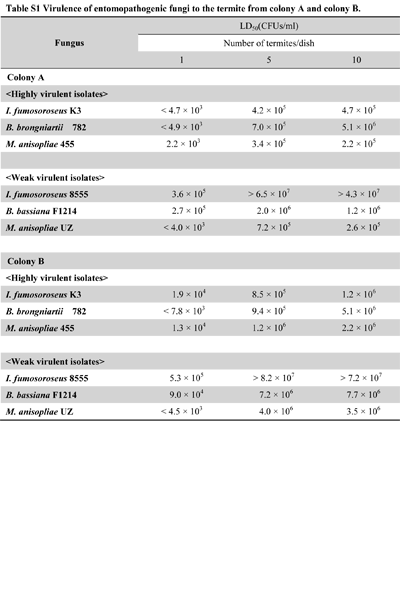

Supplement: Table S1 — Virulence of entomopathogenic fungi to the termite from colony A and colony B. (TIF) [file pone.0047412.s004.tif]

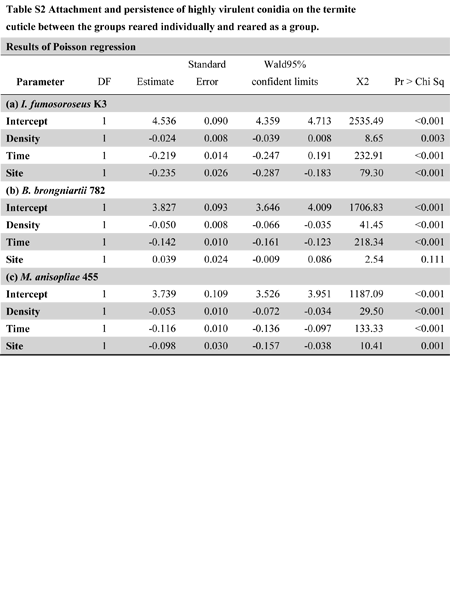

Supplement: Table S2 — Attachment and persistence of highly virulent conidia on the termite cuticle between the groups reared individually and reared as a group. (TIF) [file pone.0047412.s005.tif]

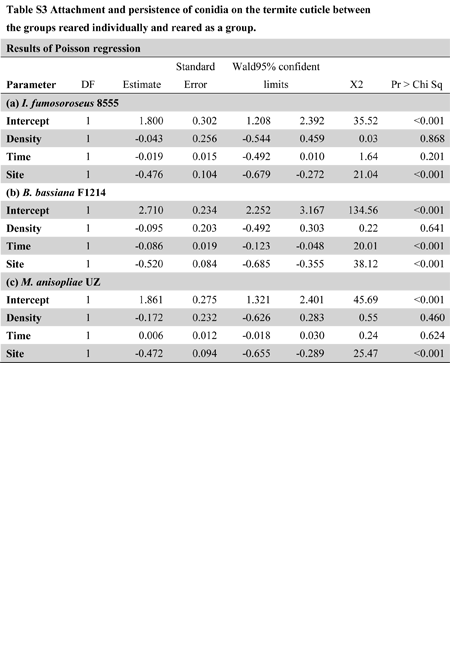

Supplement: Table S3 — Attachment and persistence of conidia on the termite cuticle between the groups reared individually and reared as a group. (TIF) [file pone.0047412.s006.tif]

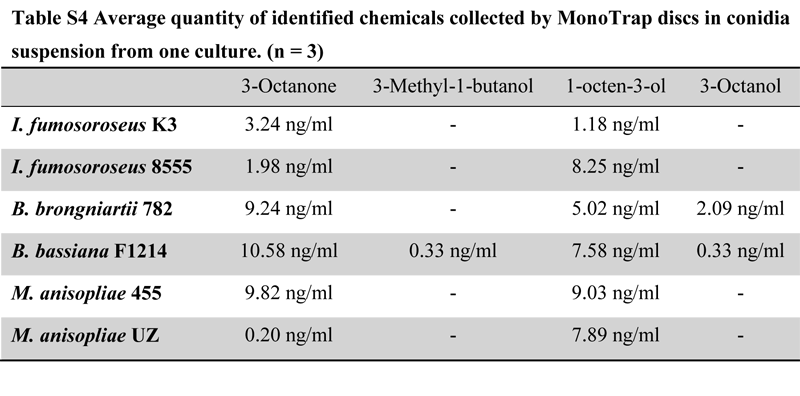

Supplement: Table S4 — Average quantity of identified chemicals collected by MonoTrap discs in conidia suspension from one culture. (TIF) [file pone.0047412.s007.tif]
